# The Association between Dietary Intakes of Vitamins and Minerals with Tinnitus

**DOI:** 10.3390/nu16152535

**Published:** 2024-08-02

**Authors:** Diana Tang, Giriraj S. Shekhawat, George Burlutsky, Paul Mitchell, Bamini Gopinath

**Affiliations:** 1 Macquarie University Hearing, Department of Health Sciences, Macquarie University, Sydney, NSW 2109, Australia; george.burlutsky@mq.edu.au (G.B.); bamini.gopinath@mq.edu.au (B.G.); 2 College of Education, Psychology, and Social Work, Flinders University, Adelaide, SA 5001, Australia; giriraj.shekhawat@flinders.edu.au; 3 Tinnitus Research Initiative, 93053 Regensburg, Germany; 4 Centre for Vision Research, Westmead Institute for Medical Research, Westmead, NSW 2145, Australia; paul.mitchell@sydney.edu.au; 5 Department of Ophthalmology, University of Sydney, Camperdown, NSW 2006, Australia

**Keywords:** tinnitus, iron, zinc, dietary intake, incidence, prevalence

## Abstract

Background: Tinnitus is the phantom perception of sound in the ears or head which may result from inflammation of the auditory pathway. A healthy diet consisting of a range of vitamins and minerals may be protective against tinnitus. This study aims to determine the association between intakes of dietary vitamins and minerals and the prevalence and incidence of tinnitus over 10 years. Methods: In this longitudinal cohort study of 2947 participants (aged ≥ 50 years), 935 (32%) cases of tinnitus were identified and included in prevalence analyses. The remaining 2012 participants were followed to establish 10-year incidence of tinnitus. A validated semi-quantitative food frequency questionnaire was used to determine intakes of dietary vitamins and minerals. Results: No significant associations with tinnitus prevalence were found. However, iron and zinc were significantly associated with incident tinnitus. There was a 44% (multivariate-adjusted HR: 1.44, 95% CI: 1.07–1.93) increased risk of developing incident tinnitus over 10 years with lower zinc intakes and a 35% increased risk with lower iron intakes (multivariate-adjusted HR: 1.35, 95% CI: 1.00–1.80). Conclusion: Higher intakes of zinc and iron were significantly associated with lower tinnitus risk. Due to a lack of comparable high-quality data, future research studies should include robust study designs.

## 1. Introduction

Tinnitus has a global prevalence of 14.4% in all adults, increasing to 23.6% in older adults [1]. It is characterised by a phantom perception of sound such as ringing or buzzing in the ears or head that can be constant or intermittent. Although most people with tinnitus are not bothered by its symptoms, 120 million people, of which most are older adults, consider their tinnitus a significant problem [1]. Severe tinnitus has debilitating quality of life impacts causing poor sleep, difficulties with attention, and feelings of frustration, anxiety, and depression [2]. While the pathophysiology of tinnitus remains unclear, strategies to prevent and cure the condition remain elusive. Currently, available treatment options mainly focus on the relief and management of tinnitus symptoms [2]. These treatments include pharmacotherapy, education, counselling, cognitive behavioural therapy, and sound therapy [2]. 

There are many exposures and aetiologies that explain the occurrence of tinnitus, e.g., genetic factors, hearing loss, noise exposure, and head injury. [1] Another potential pathway is inflammation, as suggested by a recent systematic review [3]. As age is a significant predictor of both inflammation [4] and tinnitus [1,5], strategies to promote healthy ageing may be an alternative treatment option or preventative solution. According to the World Health Organisation, ‘healthy ageing’ is defined as “more than just the absence of disease; it is the process of developing and maintaining the functional ability that enables wellbeing in older age” [6]. Diet is a domain that contributes to the concept of ‘healthy ageing’ [7] and thus may play a role in the development and management of tinnitus. The growing interest in diet and tinnitus is evidenced by the various population-based studies found in the literature [8,9,10,11,12,13,14]. However, there is a lack of longitudinal evidence on the associative links between dietary intakes of vitamins and minerals and tinnitus in ageing adults. Vitamins and minerals are essential for health due to their role in various metabolic pathways and their antioxidant and anti-inflammatory properties [15]. 

This study aims to address this knowledge gap by reporting on the associations between these essential nutrients and the prevalence and 10-year incidence of tinnitus in a population-based cohort of Australians aged 50 years and older. Findings from this novel study will add to the literature to understand whether diet is a modifiable lifestyle risk factor of tinnitus.

## 2. Materials and Methods

### 2.1. Study Participants

The Blue Mountains Hearing Study (BMHS) was conducted in accordance with the Declaration of Helsinki and was approved by The University of Sydney Human Research Ethics Committee (reference: HREC 9826). The methodology of the BMHS has been previously published [9]. The baseline population of our study comprised 2956 participants aged 49 years and older from the Blue Mountains Eye and Hearing Study II (1997–1999). Of these participants, 2947 participants had data on tinnitus symptoms and dietary intakes. Data on tinnitus symptoms consisted of the presence of tinnitus as determined by the following question administered by an audiologist: “Have you experienced any prolonged ringing, buzzing, or other sounds in your ears or head within the past year that is lasting for five minutes or longer?” In addition, data on tinnitus risk factors including the presence of any hearing loss and a history of ear infection and dizziness symptoms were also collected. To evaluate incident tinnitus, we defined this as participants who did not report the presence of tinnitus symptoms at baseline but reported it at the 5- or 10-year follow-ups. 

### 2.2. Dietary Intakes of Vitamins and Minerals

A food frequency questionnaire (FFQ) was used to calculate the self-reported dietary intakes of dietary vitamins and minerals across 145 food items. This FFQ was validated against three four-day weighed food records completed by a random subsample of BMES participants (*n* = 79) [16]. From this validity study, most nutrient correlations were between 0.50 and 0.60 for energy-adjusted intakes [16]. To complete the FFQ, our study participants were asked to indicate their consumption of each food item by selecting one of the nine frequency options, ranging from ‘Never’ to ‘4+ per day’. Beside each food item was a reference portion size. FFQ data were then linked to the Australian food composition tables from 1990 (formerly NUTTAB 90) [17], which report the amount of vitamins and minerals within each food item. The vitamins or minerals that were assessed were as follows: α-carotene; β-carotene; β-cryptoxanthin; combined lutein and zeaxanthin; lycopene; vitamins A (retinol equivalents), C, and E; and iron and zinc as cofactors for antioxidant enzymes. 

Overall dietary intakes of vitamins and minerals were calculated by summing the amount consumed by the participants for each food item. Dietary intakes were reported in mg/d.

### 2.3. Statistical Analysis

SAS statistical software (SAS Institute, Cary, NC, USA) version 9.4 was used to conduct the statistical analyses. Descriptive statistics were used to describe the study population who developed tinnitus over the 10 years. To assess the cross-sectional association between dietary intakes (expressed as quintiles of intake) and the prevalence of tinnitus, a logistic regression analysis was conducted to determine the odds ratios with corresponding 95% confidence intervals (CIs). Discrete-time proportional hazard models were used to evaluate their associations with incident tinnitus over 10 years. Namely, discrete-time survival models are applied to person–period data to predict the hazard of experiencing an event (in this case, tinnitus) in pre-specified time intervals, i.e., for this study, between baseline and a 5-year follow-up or between the 5- and 10-year follow-ups. We used proc phreg with option ties = discrete. This option in SAS replaces the proportional hazard model with the discrete logistic model. Dietary intakes were energy-adjusted; the results are presented with age/sex adjustments and multivariate adjustments accounting for age, sex, dizziness symptoms, middle ear infections, and any hearing loss. Based on initial analyses, only iron and zinc intakes showed significant associations and further analyses focused on these nutrients, including comparisons between the lowest quintile of intake to higher intakes. Statistical significance was indicated by a *p* value < 0.05.

## 3. Results

Participant characteristics are shown in Table 1. In this cohort, there were more women (57%), and the mean age was 67.4 years. More than half had a history of hypertension (53.4%), and approximately one-third had dizziness symptoms (36.5%) and hearing loss (32.9%). Other comorbidities were less prevalent. Of the 2947 participants at baseline, 935 (31.7%) presented with tinnitus. Among these participants, there was no association between the dietary intakes of vitamins and minerals and prevalent tinnitus. For the remaining 2012 participants who did not present with tinnitus at baseline, 1109 (55%) had 10-year follow-up data. 

As shown in Table 2, the overall trend of association for both nutrients was not significant, but some significance was observed with higher intakes. Further analyses shown in Table 3 compared iron intakes ≤ 9.51 mg/d (Quintile 1; Q1) versus >9.51 mg/d (Quintiles 2 to 5, Q2–5, combined). These findings showed that lower intakes of iron significantly increased the risk of incident tinnitus by 35% (hazard ratio, HR, 1.35, 95% confidence intervals, CIs, 1.00–1.90). Similarly, lower intakes of zinc (≤8.48 mg/d, Q1) versus intakes greater than 8.48 mg/d (Q2, Q3, Q4, and Q5 combined) significantly increased the risk of incident tinnitus by 44% (HR 1.44, 95% CI 1.07–1.93). 

## 4. Discussion

This is the first longitudinal study to explore the associative links between dietary intakes of vitamins and minerals and the prevalence and incidence of tinnitus in an older adult population. Our findings suggest that intakes of iron ≤ 9.51 mg/d and of zinc ≤ 8.48 mg/d significantly increased the risk of incident tinnitus over 10 years by 35% and 44%, respectively, compared to those who consumed higher intakes. In comparison to the equivalent recommended dietary intake (RDI) for this age group in Australia, the cut-off points for the lowest quintile of iron intake (≤9.51 mg/d) exceeds the RDI for iron (8 mg/d) [18] while the lowest intake for zinc (≤8.48 mg/d) aligns with the RDI for women (8 mg/d) but falls short of the recommendations for men (14 mg/d) [19]. Therefore, RDI targets may not reflect the required intakes needed to mitigate the risk of conditions like tinnitus.

In our study, tinnitus prevalence was not significantly associated with vitamin and mineral intake. This contradicts findings from other cross-sectional, population-based studies; for example, Dawes et al. identified and reported that higher intakes of iron had a significant negative impact, increasing the odds of tinnitus among 40- to 69-year-old UK adults [8]. Notably, the authors of this study acknowledged that this finding contradicted their other finding that a high-protein diet, which consists of iron-rich sources, was significantly associated with reduced odds of tinnitus [8]. This also contradicts existing research interest regarding iron-deficiency anaemia and tinnitus risk. This interest is based on the understanding that iron-deficiency anaemia describes a lack of healthy haemoglobin in red blood cells and insufficient oxygen circulation around the body. Poor circulation, and thus reduced blood supply, to the inner ear, is hypothesised to increase susceptibility to ischemic damage leading to impaired inner ear functioning [20,21].

In the literature, most studies focus on the impacts of iron-deficiency anaemia on hearing loss [20,22,23,24] with limited studies exploring the association with tinnitus [21,25]. A study conducted in Korea explored the relationship between iron-deficiency anaemia and tinnitus [21]. When analysing data from the Korea National Health and Nutrition Examination Survey (2010–2011), a nonsignificant association (*p* = 0.064) was reported [21]. However, in the second part of their study, retrospective analyses of clinical patient data showed favourable tinnitus outcomes within one month after commencing treatment for the anaemia with iron supplementation and blood transfusions [21]. This suggests that there may be an indirect relationship between adequate iron status and tinnitus risk, but this would need further confirmation by other large cohort studies.

The second significant finding from our study is that an association between lower dietary zinc intake and a greater risk of incident tinnitus exists. Namely, participants who had a zinc intake of ≤8.48 mg/d (Q1) compared to those who had higher intakes (>8.48 mg/d, in Q2–Q5) had a 44% greater risk of incident tinnitus over 10 years. Given that ours is an observational study, we can only posit potential underlying mechanisms for this link. One potential mechanism is that zinc is involved in the cochlear pathology and the synapses of the auditory system [26]. As zinc plays a key role in various cellular processes and pathways and has antioxidant and anti-inflammatory properties [27], lower intakes of zinc may increase the susceptibility of the auditory system to the effects of inflammation. This is based on findings from a recent systematic review which suggested that inflammation of the auditory pathway increases excitatory neurotransmission and decreases inhibitory ones resulting in neuroplasticity causing chronic tinnitus [3]. 

While our study and others warrant further research into zinc supplementation as a treatment option for tinnitus, the evidence from a 2016 Cochrane review does not show a significant link between zinc supplementation and tinnitus risk [26]. The lack of significant association in this review could be due to the inclusion of a small number of eligible studies that were of low quality, as well as due to heterogeneity related to tinnitus, where patients can experience differences in their tinnitus from the perception of symptoms, associated distress, and the response to treatment [28]. To overcome these challenges, higher-quality research evidence, including those from large population-based longitudinal studies, is needed to draw accurate and representative conclusions. The potential for a beneficial association has been reported in a more recent case–control study conducted in 2019 [29]. In this study, twenty adults with tinnitus and 20 healthy controls completed a test battery including medical history assessment, otoscopic and audiologic evaluation, questionnaires, and serum zinc analyses. This study found that oral zinc supplementation elevated serum zinc levels and led to significantly improved Tinnitus Handicap Inventory total scores in 17 out of 20 (85%) patients with noise-induced tinnitus (*p* = 0.024). As more studies are conducted, another review is warranted to capture the latest evidence.

To our knowledge, intervention studies exploring the influence of dietary zinc and iron intake on tinnitus have not yet been conducted. Nutrients obtained through the diet are more potent [30], and food-focused research may yield more information by capturing the unknown influence of the food matrix [31]. This approach is needed as single-nutrient solutions (i.e., supplements) are generally linked to relative deficiencies such as those arising from restrictions of certain foods or food groups, e.g., veganism, or due to physiological changes such as pregnancy [31]. Based on current knowledge, tinnitus is not linked to a nutrient deficiency. Food or dietary pattern-related research may lead to a better understanding of the role of iron and zinc for tinnitus especially since evidence from our study, which captured intakes through the diet, identified significant associations.

### Strengths and Limitations

This study includes a representative population of older adults and the use of a validated food questionnaire to capture usual dietary intake. Further, the approach to analysis in this study, that is, using the discrete-time proportional hazard model is ideal for determining the incidence of a condition due to its flexibility with discrete time intervals, effective handling of censored data, the accommodation of time-dependent covariates, and proportional hazards assumption. However, we also acknowledge the following limitations: Firstly, the sample size of incident tinnitus over 10 years is small. This may have contributed to the lack of significant findings with other vitamins and minerals. Secondly, the FFQ captures self-reported usual intake in the last 12 months and reported intakes may vary from actual intakes. Thirdly, recall bias may be apparent in the identification of tinnitus which was determined from a single question. Specifically, we did not ascertain tinnitus with objective methods such as pitch matching, loudness matching testing, and masking, which may have led to under- or over-reporting of tinnitus symptoms. As a result of these limitations, our findings do not suggest a causal relationship between low intakes of iron and zinc and incident tinnitus. Further research is recommended, including interventional studies, to confirm this association.

## 5. Conclusions

Due to the expected increase in tinnitus prevalence as our population continues to age, solutions are needed to reduce the burden of its severe symptoms. This is the first longitudinal study to report that low dietary intakes of iron and zinc can potentially increase the risk of developing tinnitus over 10 years in older adults. However, this association was marginally significant and therefore, conclusions should be drawn with caution. As the current literature lacks robust data, particularly on links between food-based sources of these nutrients and tinnitus risk, future research efforts should focus on high-quality study designs, such as those from large cohort studies and randomised controlled trials, to confirm our findings.

## Figures and Tables

**Table 1:** Participant characteristics at baseline.

Women, *n* (%)	1679 (57.0)
Age, mean (SD)	67.4 (9.2)
Any hearing loss, *n* (%)	965 (32.9)
Dizziness symptoms, *n* (%)	1009 (36.5)
Middle ear infection, *n* (%)	315 (11.1)
History of type 2 diabetes, *n* (%)	306 (11.3)
History of CVD, *n* (%)	539 (18.4)
History of hypertension, *n* (%)	1567 (53.4)
Obesity (BMI ≥ 30 kg/m^2^), *n* (%)	700 (18.4)
Tinnitus, *n* (%)	935 (31.7)

**Table 2:** Association between dietary iron and zinc intake and 10-year incidence of tinnitus presented as hazard ratios (HRs) and 95% confidence intervals (CIs).

Nutrient *	*n* at Risk/Cases	Any Tinnitus	
		HR (95% CI) ^a^	HR (95% CI) ^b^
Iron, mg/d			
Q1 (2.11–9.51)	203/93	1.0 (reference)	1.0 (reference)
Q2 (9.52–11.50)	223/95	0.87 (0.61–1.23)	0.83 (0.57–1.19)
Q3 (11.51–13.54)	222/81	0.69 (0.48–0.99)	0.67 (0.46–0.98)
Q4 (13.55–16.14)	233/88	0.74 (0.52–1.05)	0.78 (0.54–1.12)
Q5 (16.15–37.5)	228/89	0.75 (0.53–1.07)	0.70 (0.48–1.02)
*p* value for trend	-	0.09	0.07
Zinc, mg/d			
Q1 (1.97–8.48)	204/99	1.0 (reference)	1.0 (reference)
Q2 (8.49–10.45)	235/88	0.66 (0.47–0.94)	0.72 (0.50–1.04)
Q3 (10.46–12.29)	229/77	0.55 (0.38–0.78)	0.54 (0.37–0.79)
Q4 (12.30–14.56)	220/95	0.84 (0.59–1.18)	0.82 (0.57–1.18)
Q5 (14.57–43.38)	221/87	0.70 (0.49–0.99)	0.74 (0.51–1.07)
*p* value for trend	-	0.21	0.26

CI—confidence interval; HR—hazard ratio; Q—quintile. * Adjusted for energy intakes. ^a^ Adjusted for age and sex. ^b^ Adjusted for age, sex, dizziness symptoms, middle ear infections, and any hearing loss.

**Table 3:** The association between the lowest versus higher intakes of iron and zinc and the 10-year incidence of tinnitus presented as hazard ratios (HRs) and 95% confidence intervals (CIs).

Nutrient *	*n* at Risk/Cases	Any Tinnitus	
		HR (95% CI) ^a^	HR (95% CI) ^b^
Iron, mg/d			
Lower intake ≤ 9.51 mg (Q1)	203/93	1.31 (0.99–1.74)	1.35 (1.00–1.80)
Higher intakes > 9.51 mg (Q2–Q5)	906/353	1.0 (reference)	1.0 (reference)
Zinc, mg/d			
Lower intake ≤ 8.48 mg (Q1)	204/99	1.48 (1.12–1.95)	1.44 (1.07–1.93)
Higher intakes > 8.48 mg (Q2–Q5)	905/347	1.0 (reference)	1.0 (reference)

CI—confidence interval; HR—hazard ratio; Q—quintile. * Adjusted for energy intakes. ^a^ Adjusted for age and sex. ^b^ Adjusted for age, sex, dizziness symptoms, middle ear infections, and any hearing loss.

## Data Availability

The data presented in this study are available upon request from the corresponding author. The data are not publicly available due to privacy restrictions.
